# 296. MDR Risk Index: Multidrug-Resistant Bacteria Probability Score for General Hospitals

**DOI:** 10.1093/ofid/ofae631.086

**Published:** 2025-01-29

**Authors:** Edna M M Leite, Esther Camargos, Lilian Aguiar, Wagner Luiz De Oliveira, Bráulio R G M Couto

**Affiliations:** Hospital Risoleta Tolentino Neves, Belo Horizonte, Minas Gerais, Brazil; Hospital Risoleta Tolentino Neves - HRTN, Belo Horizonte, Minas Gerais, Brazil; Hospital Risoleta Tolentino Neves - HRTN, Belo Horizonte, Minas Gerais, Brazil; Hospital Risoleta Tolentino Neves - HRTN, Belo Horizonte, Minas Gerais, Brazil; AMECI – Associação Mineira de Epidemiologia e Controle de Infecções, Belo Horizonte, Minas Gerais, Brazil

## Abstract

**Background:**

This research endeavors to shed light on the intricate relationship between hospitalization dynamics and the probability of acquiring multidrug-resistant bacteria (MDR) by introducing a predictive tool, the Multidrug-Resistant Bacteria Probability Score.

**Figure 1.**
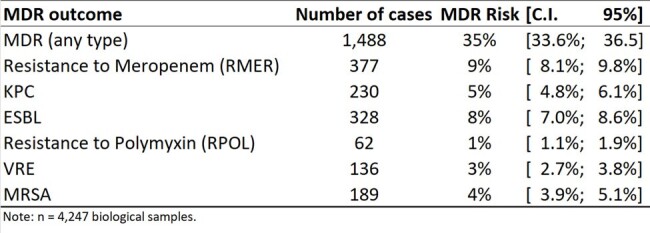
Estimated Risk of MDR (Multidrug-Resistant Bacteria): Point Estimates and 95% Confidence Intervals.

**Methods:**

We processed cultures in the hospital laboratory using automated systems and/or microdilution methods, adhering to the BR-cast guidelines. We utilized a logistic regression model to analyze four predictive variables for MDR: length of hospitalization until the collection of biological material (days), previous hospitalization (yes/no), duration of previous hospital stays (days), and admission to the Intensive Care Unit (ICU) (yes/no). The assessment focused on 7 outcomes: MDR (any type), resistance to Meropenem (RMER), production of Klebsiella pneumoniae carbapenemase (KPC), production of Extended Spectrum Beta-Lactamase (ESBL), resistance to Polymyxin (RPOL), Vancomycin-resistant Enterococci (VRE), and Methicillin-resistant Staphylococcus aureus (MRSA).

**Figure 2.**
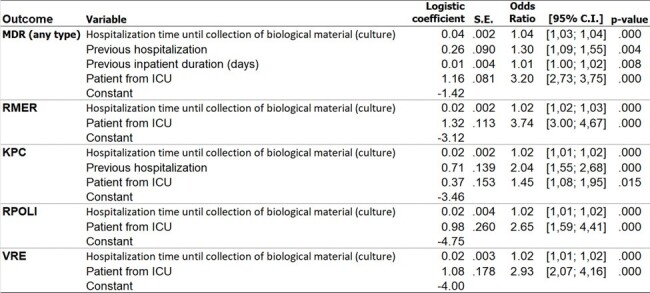
Logistic Regression Models Estimating MDR Risk: Results for Each Type of MDR Bacteria.

**Results:**

A total of 4,247 biological samples were collected from Nov/2022 to Nov/2023: Urine (46%), Blood (12%), Tissue Fragments (11%), Bronchoalveolar Lavage (6%), Perianal Swabs (6%), Tracheal Aspirates (4%), Nasal Swabs (3%), Abdominal Fluid (2%), Tips of Central or Umbilical Catheters (2%), Wound Secretions (1%), and Other (3%). Approximately one-third (35%) of the samples were identified as MDR. Unadjusted MDR risk: 9% RMER, 5% KPC, 8% ESBL, 1% RPOL, 3% VRE, and 4% MRSA (Fig. 1). From the seven outcomes modeled, it was possible to successfully predict five of them (Fig. 2), yielding good results in terms of predictive capability (Fig. 3). However, the modeling was unsuccessful for MRSA and ESBL. Figure 4 shows simulations of MDR risk.

**Figure 3.**
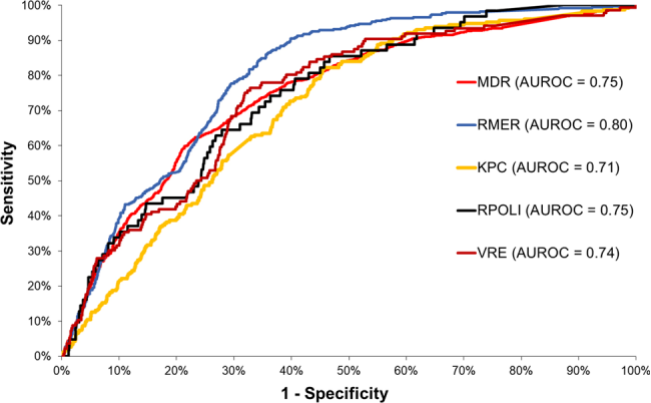
ROC Curves for the Five Successful Models Predicting Multidrug-Resistant Bacteria (MDR).

**Conclusion:**

The duration of hospitalization prior to the collection of biological material emerges as a crucial metric, encapsulating the temporal dimension of patient exposure to potential sources of infection. All five predictive models can be effectively integrated into the patient’s electronic health record. This integration enables the timely prediction of multidrug-resistant (MDR) infections, facilitating the implementation of targeted preventive measures based on the assessed risk level.

**Figure 4.**
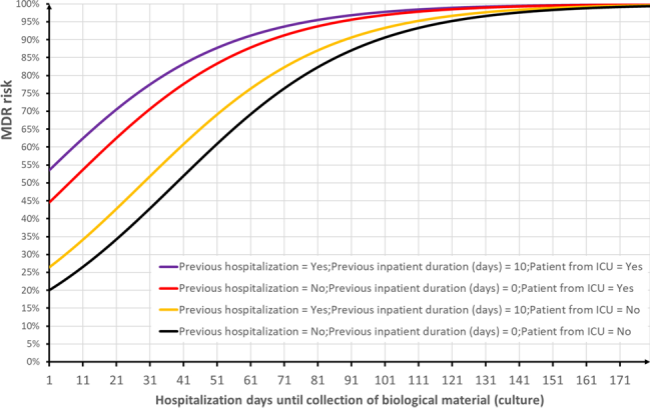
Simulation of MDR Risk Evolution Over Hospitalization Time: Curves Representing MDR Risk by Patient Type

**Disclosures:**

**All Authors**: No reported disclosures

